# Draft Genome Sequence of Lampenflora *Chlorobium limicola* Strain Frasassi in a Sulfidic Cave System

**DOI:** 10.1128/genomeA.00357-16

**Published:** 2016-05-12

**Authors:** Muammar Mansor, Jennifer L. Macalady

**Affiliations:** Department of Geosciences, Pennsylvania State University, University Park, Pennsylvania, USA

## Abstract

The draft genome sequence of *Chlorobium limicola* strain Frasassi was assembled from metagenomic sequencing of a green mat in an artificially lighted aquarium inside the Frasassi caves in Italy. The genome is 2.08 Mbp in size and contains the necessary genes for anoxygenic photosynthesis and CO_2_ fixation.

## GENOME ANNOUNCEMENT

Historic cave sites face defamation problems from growth of photosynthetic microorganism communities growing under artificial lights termed lampenflora (1). In 2010, an aquarium with dim lighting (6.5 µmol photon/m^2^/s) was mounted inside the tourist-accessible Frasassi cave system, Italy. Water from the deep sulfidic groundwater table is periodically pumped into the aquarium to maintain stratified conditions. Since 2012, a 1-cm-thick green mat has been perennially present in the tank and was sampled for genetic analyses.

We extracted DNA using the PowerBiofilm DNA isolation kit (Mo Bio Laboratories, Inc., Carlsbad, CA) and sequenced the DNA using Illumina HiSeq PE150 (San Diego, CA). The reads were trimmed with Trimmomatic (2) and assembled with IDBA (3). Metagenomic binning and preliminary identification were performed using Metawatt (4) based on tetranucleotide composition. A putative *Chlorobium* bin was split into a high-coverage and a low-coverage bin, discernible by the existence of two clusters in a plot of coverage versus the G+C content. The high-coverage putative *Chlorobium* bin was annotated with RAST (5, 6), KEGG (7, 8), and BLAST, with an e-value cutoff of 1e-5.

Genome completeness was estimated at 95% based on genes for ribosomal proteins and tRNA-synthetases. The total genome length is 2.08 Mbp, captured in 258 contigs, with an *N*_50_ of 16,277 bp. Coverage is approximately 3,000×. Phylogenetic trees based on 16S rRNA sequences support the classification of this microorganism as a strain of *Chlorobium limicola* (99.9% similarity), designated strain Frasassi.

*C. limicola* strain Frasassi has the genes necessary for performing anoxygenic photosynthesis using reduced S species. Oxidation of sulfide can be catalyzed by SqrD (9), while elemental sulfur oxidation can be catalyzed by rDsrAB. Strain Frasassi also contains the complete pathway for the synthesis of bacteriochlorophyll *c* (BChl *c*) and the carotenoid chlorobactene and for carbon dioxide fixation through the reverse tricarboxylic acid (TCA) cycle.

The genome of strain Frasassi is about 0.7 Mbp smaller than the genome of the species type strain, *C. limicola* DSM 245 (10). In total, 2,697 genes are absent in strain Frasassi relative to DSM 245. Thirty-three percent of those genes encode hypothetical proteins or proteins with unknown functions. The other missing genes include the Sox pathway genes (for oxidizing thiosulfate), the *dsrMKJOP* cluster, vitamin B_12_ synthesis, and some DNA repair and replication and sugar biosynthesis genes. Lastly, strain Frasassi has clustered regularly interspaced short palindromic repeat (CRISPR) type III, while *C. limicola* DSM 245 has CRISPR type I.

The unexpected appearance of an obligate phototroph and anaerobe with no known resting stages in a tourist cave is surprising. Further genetics-enabled biogeographic studies of this strain and strains retrieved from the sunlit environment outside the cave system have the potential to reveal the origin of the lampenflora population and the time scale and mode of its dispersal to the artificial aquarium.

### Nucleotide sequence accession numbers.

The genome sequence of *C. limicola* strain Frasassi has been deposited in DDBJ/EMBL/GenBank under the accession no. LMBR00000000. The version described here is version LMBR01000000.

## References

[1] Mulec J, Kosi G 2009 Lampenflora algae and methods of growth control. J Cave Karst Stud 71:109–115.

[2] Bolger AM, Lohse M, Usadel B 2014 Trimmomatic: a flexible trimmer for Illumina sequence data. Bioinformatics 30:2114–2120. doi:10.1093/bioinformatics/btu170. 24695404 PMC4103590

[3] Peng Y, Leung HCM, Yiu SM, Chin FYL 2012 IDBA-UD: a *de novo* assembler for single-cell and metagenomic sequencing data with highly uneven depth. Bioinformatics 28:1420–1428. doi:10.1093/bioinformatics/bts174. 22495754

[4] Strous M, Kraft B, Bisdorf R, Tegetmeyer HE 2012 The binning of metagenomic contigs for microbial physiology of mixed cultures. Front Microbiol 3:410. doi:10.3389/fmicb.2012.00410. 23227024 PMC3514610

[5] Aziz RK, Bartels D, Best AA, DeJongh M, Disz T, Edwards RA, Formsma K, Gerdes S, Glass EM, Kubal M, Meyer F, Olsen GJ, Olson R, Osterman AL, Overbeek RA, McNeil LK, Paarmann D, Paczian T, Parrello B, Pusch GD 2008 The RAST server: Rapid Annotations using Subsystems Technology. BMC Genomics 9:75. doi:10.1186/1471-2164-9-75. 18261238 PMC2265698

[6] Overbeek R, Olson R, Pusch GD, Olsen GJ, Davis JJ, Disz T, Edwards RA, Gerdes S, Parrello B, Shukla M, Vonstein V, Wattam AR, Xia F, Stevens R 2014 The SEED and the rapid annotation of microbial genomes using subsystems technology (RAST). Nucleic Acids Res 42:D206–D214. doi:10.1093/nar/gkt1226. 24293654 PMC3965101

[7] Kanehisa M, Goto S 2000 KEGG: Kyoto Encyclopedia of Genes and Genomes. Nucleic Acids Res 28:27–30. doi:10.1093/nar/28.1.27. 10592173 PMC102409

[8] Kanehisa M, Goto S, Kawashima S, Okuno Y, Hattori M 2004 The KEGG resource for deciphering the genome. Nucleic Acids Res 32:D277–D280. doi:10.1093/nar/gkh063. 14681412 PMC308797

[9] Gregersen LH, Bryant DA, Frigaard N-U 2011 Mechanisms and evolution of oxidative sulfur metabolism in green sulfur bacteria. Front Microbiol 2:116. doi:10.3389/fmicb.2011.00116. 21833341 PMC3153061

[10] Bryant DA, Liu Z, Li T, Zhao F, Costas AMG, Klatt CG, Ward DM, Frigaard N-U, Overmann J 2012 Comparative and functional genomics of anoxygenic green bacteria from the taxa *Chlorobi*, *Chloroflexi*, and *Acidobacteria*, p 47–102. *In* Burnap RL, Vermaas WFJ (ed), Advances in photosynthesis and respiration, volume 33: functional genomics and evolution of photosynthetic systems. Springer, New York, NY.

